# Supplementary material for: A Global Screen for Assembly State Changes of the Mitotic Proteome by SEC-SWATH-MS
Source: Cell Syst. 2020 Feb 26;10(2):133–155.e6. doi: 10.1016/j.cels.2020.01.001 (PMC7042714; doi:10.1016/j.cels.2020.01.001)

Q9GZT8 | NIF3L_HUMAN | NIF3L1 ALS2CR1 MDS015 My018
Monomer MW [kDa]: 41.968 Monomer expected elution fraction: 48

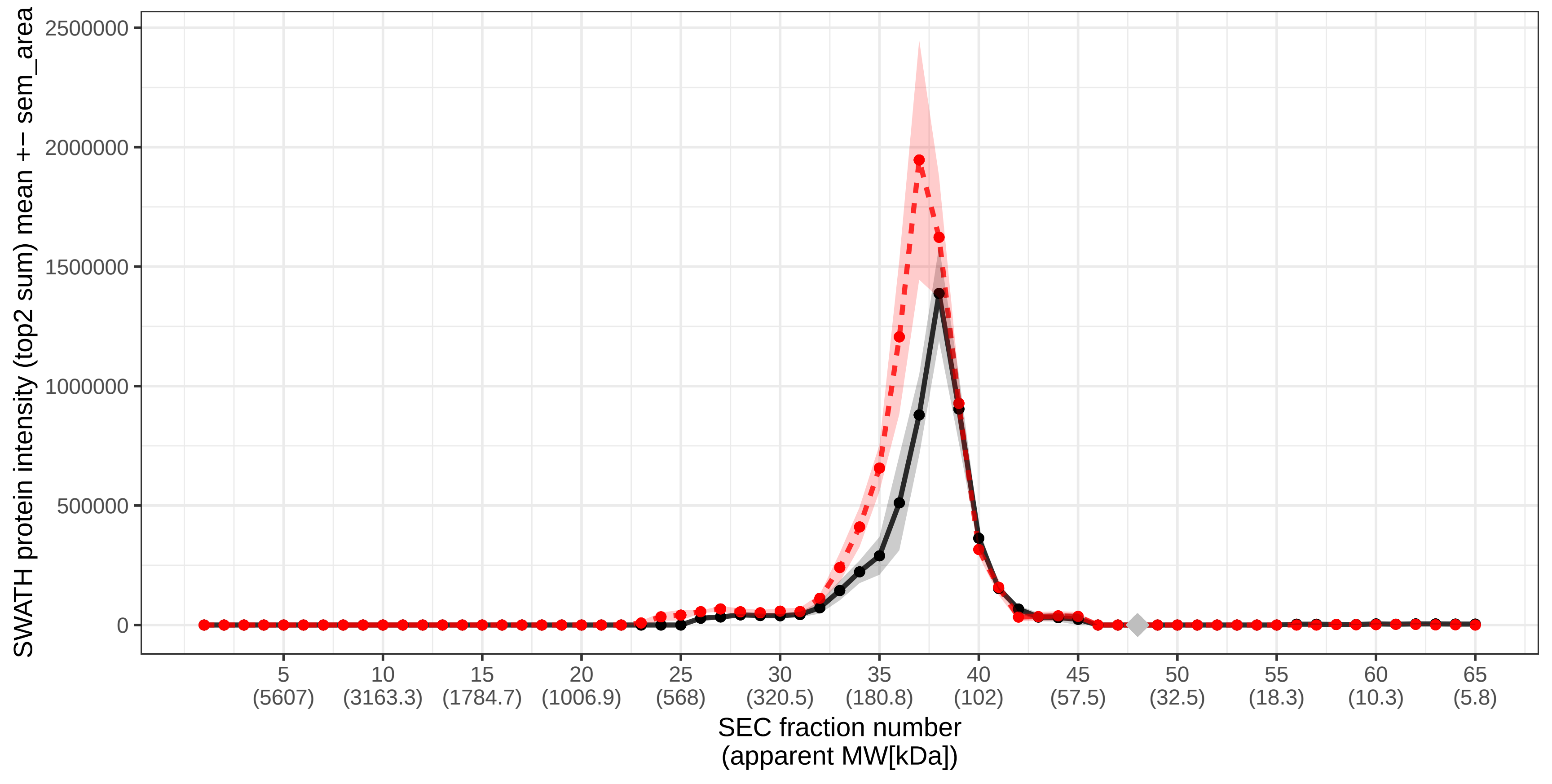

Supplement: Data S1. SEC-SWATH-MS Protein Chromatograms, Related to Figure 1 [file mmc6.zip › SECchrom_Q9GZT8_NIF3L_HUMAN_NIF3L1_ALS2CR1_MDS015_My018.pdf]
